# Use of glucocorticoids in patients with acute respiratory distress syndrome: a meta-analysis and trial sequential analysis

**DOI:** 10.1186/s40560-020-00464-1

**Published:** 2020-06-30

**Authors:** Yazan Zayed, Mahmoud Barbarawi, Esraa Ismail, Varun Samji, Josiane Kerbage, Fatima Rizk, Mohammad Salih, Areeg Bala, Michele Obeid, Smit Deliwala, Sherry Demian, Ibrahim Al-Sanouri, Raju Reddy

**Affiliations:** 1 Department of Internal Medicine, Hurley Medical Center/Michigan State University, One Hurley Plaza, Suite 212, Flint, MI 48503 USA; 2 College of Human Medicine, Michigan State University, East Lansing, MI USA; 3 Department of Anesthesia, Lebanese University, Beirut, Lebanon; 4 College of Osteopathic Medicine, Michigan State University, East Lansing, MI USA; 5 Department of Pulmonary and Critical Care, Hurley Medical Center/Michigan State University, Flint, MI USA; 6 Department of Pulmonary and Critical Care Medicine, University of Florida, Gainesville, FL USA

**Keywords:** Glucocorticoids, Corticosteroids, Acute respiratory distress syndrome, ARDS, Meta-analysis

## Abstract

**Background:**

Acute respiratory distress syndrome (ARDS) is a common and disabling disease with high rates of mortality and morbidity. The role of steroids in treating ARDS remains controversial. We aim to examine the evidence behind using glucocorticoids in the management of ARDS from the available studies.

**Methods:**

We performed a literature review of major electronic databases for randomized controlled trials (RCTs) comparing glucocorticoids versus placebo in treating patients with ARDS. Our primary outcome was hospital mortality. Other outcomes included ICU mortality, number of ventilator-free days at day 28, incidence of nosocomial infections, and hyperglycemia. We performed a meta-analysis using a random effects model to calculate risk ratios (RR) and mean difference (MD) with their corresponding 95% confidence intervals (CI). A subsequent trial sequential analysis was performed to examine the strength of evidence and to guard against statistical type I and type II errors for our results.

**Results:**

Eight RCTs were included in the final analysis totaling of 1091 patients, with a mean age of 57 ± 16, and 56.2% were male. In our pooled analysis, use of glucocorticoids was associated with a significant reduction in hospital mortality (RR 0.79; 95% CI 0.64–0.98; *P* = 0.03) and ICU mortality (RR 0.64; 95% CI 0.42–0.97; *P* = 0.04). Furthermore, glucocorticoid use was associated with an increased number of ventilator-free days at day 28 (MD 4.06 days; 95% CI 2.66–5.45; *P* < 0.01). Regarding adverse events, glucocorticoids use was not associated with an increased risk for nosocomial infections (RR 0.82; 95% CI 0.68–1.00; *P* = 0.05); however, it was associated with an increased risk of hyperglycemia (RR 1.11; 95% CI 1.01–1.24; *P* = 0.04). In our trial sequential analysis, the required diversity-adjusted information size (sample size = 2692 patients) was not reached, and the evidence was insufficient from the available RCTs.

**Conclusion:**

Among patients with ARDS, use of glucocorticoids is associated with a significant reduction in mortality and duration of mechanical ventilation, without increased risk of hospital-acquired infections. However, based on a trial sequential analysis, these findings may be secondary to a false-positive (type I) error. Further studies are needed for a firm conclusion with guarding against possible statistical errors.

## Introduction

Acute respiratory distress syndrome (ARDS) is a common and disabling syndrome with high rates of mortality and morbidity. It affects 10% of patients admitted to intensive care units (ICUs) and almost 23% of mechanically ventilated patients. Additionally, ARDS has been found to have up to 35–45% mortality rate [1–3].

A recently published randomized controlled trial (RCT) showed a significant reduction in short-term and long-term mortality of ARDS patients who received dexamethasone within 24 h of ARDS onset [4]. In addition, an analysis involving individual patients’ data of four randomized controlled trials (RCTs) showed significant improvement in mortality and several other clinical outcomes with glucocorticoid use in ARDS patients [5]. However, their use in ARDS is still controversial, and the current society of critical care medicine guidelines have conditional recommendations for the use of glucocorticoids in patients with moderate-to-severe ARDS [6].

In this meta-analysis, we aim to examine the efficacy and safety of glucocorticoids in ARDS, as well as examine the strength of current evidence based on the available RCTs by performing a trial sequential analysis.

## Methodology

### Study design and study selection

Our study is a meta-analysis of RCTs performed according to the Preferred Reporting Items for Systematic Reviews and Meta-Analyses Protocols (PRISMA-P) 2015 Statement [7]. Literature search utilizing major electronic databases including PubMed, Cochrane library, and Embase was conducted separately and independently by two reviewers (V.S.) and (M.S.) from inception to March 2020. Articles were first screened by abstracts and titles before exclusion. Review of full texts of eligible articles was performed before final inclusion or exclusion. Mesh term used: (“acute lung injury” OR “acute respiratory distress syndrome” OR “ARDS”) AND (“glucocorticoids” OR “corticosteroid” OR “steroids” OR “methylprednisolone” OR “dexamethasone” OR “hydrocortisone” OR “prednisolone”). In addition, references of relevant articles were reviewed for possible inclusion. Any discrepancy between the two reviewers was resolved by a third author (Y.Z.).

### Inclusion and exclusion criteria

We included only RCTs that evaluated the role of glucocorticoids in the management of critically ill adult patients with established respiratory failure secondary to ARDS; ARDS was defined as acute hypoxemic respiratory failure with presence of bilateral infiltrates on chest imaging, PaO2/FiO2 < 300 without evidence of left ventricular failure or hydrostatic edema. Studies that examined prophylactic effects of glucocorticoids in patients at high risk for ARDS were excluded. In addition, we excluded studies with a high risk of bias as well as studies unavailable in English.

Two reviewers (E.I. and J.K.) extracted the data into predesigned tables independently and separately. Any discrepancy was resolved by a third reviewer (Y.Z.).

### Quality assessment

Quality assessment of the included RCTs was performed using the Cochrane Collaboration’s tool for assessing risk of bias in randomized controlled trials [8]. Random sequence generation, allocation concealment, blindness of participants and health-care personnel, blindness of outcome assessment, incomplete outcome data, selective reporting, and other biases if any were present were assessed for each of the included RCTs based on authors’ judgement.

### Outcomes

Our primary outcome was in-hospital mortality defined as mortality before hospital discharge (if in-hospital mortality was not provided, we utilized the 60-day mortality or mortality at longest follow-up duration provided by each study in order of preference). Secondary outcomes included ICU mortality and number of ventilator free days at day 28. Safety outcomes included incidence of nosocomial infections and incidence of hyperglycemia.

### Statistical analysis

Pooled risk ratios (RR) with their corresponding 95% confidence intervals (CI) for dichotomous data were calculated using the random Mantel-Haenszel method. We calculated weighted mean difference (MD) and their 95% corresponding confidence intervals for continuous variables using an inverse variance test. Heterogeneity was assessed using Cochrane Q and *I*^2^ tests. Sensitivity analysis was performed by sequential removal of trials for each outcome. In addition, we conducted a subgroup analysis based on timing of glucocorticoids administration, early (less than 7 days of ARDS onset) vs late (> 7 days of ARDS onset), severity of ARDS, and whether studies used lung protective ventilations or not. Further meta-regression analyses were performed based on the study-level covariates [age, duration of glucocorticoids treatment (days), daily dose of glucocorticoid equivalent to prednisone, age, mean positive end-expiratory pressure, partial pressure of arterial oxygen to fractional inspired oxygen (PaO2/FiO2)]. Revman v5.3 windows and the Comprehensive Meta-Analysis v3 software were used for the analysis.

### Trial sequential analysis

To examine the strength of our results, we applied trial sequential analysis (TSA) boundaries to the meta-analysis to guard against the risk of false-positive (type I error) or false-negative (type II error) results [9]. We performed our analysis to maintain an overall two-sided type I error at 5% and to provide 80% power to calculate the diversity-adjusted information size in order to examine if the conclusion is sufficient or if further studies are needed to detect 20% relative risk reduction (RRR) of hospital and ICU mortality between the two groups. Further analyses were performed to calculate sample size required for 15% and 25% relative risk reduction of mortality. TSA software, Copenhagen Trial Unit, version 0.9.5.10 Beta was used to conduct the analysis.

## Results

### Summary of included studies

**Fig. 1:**
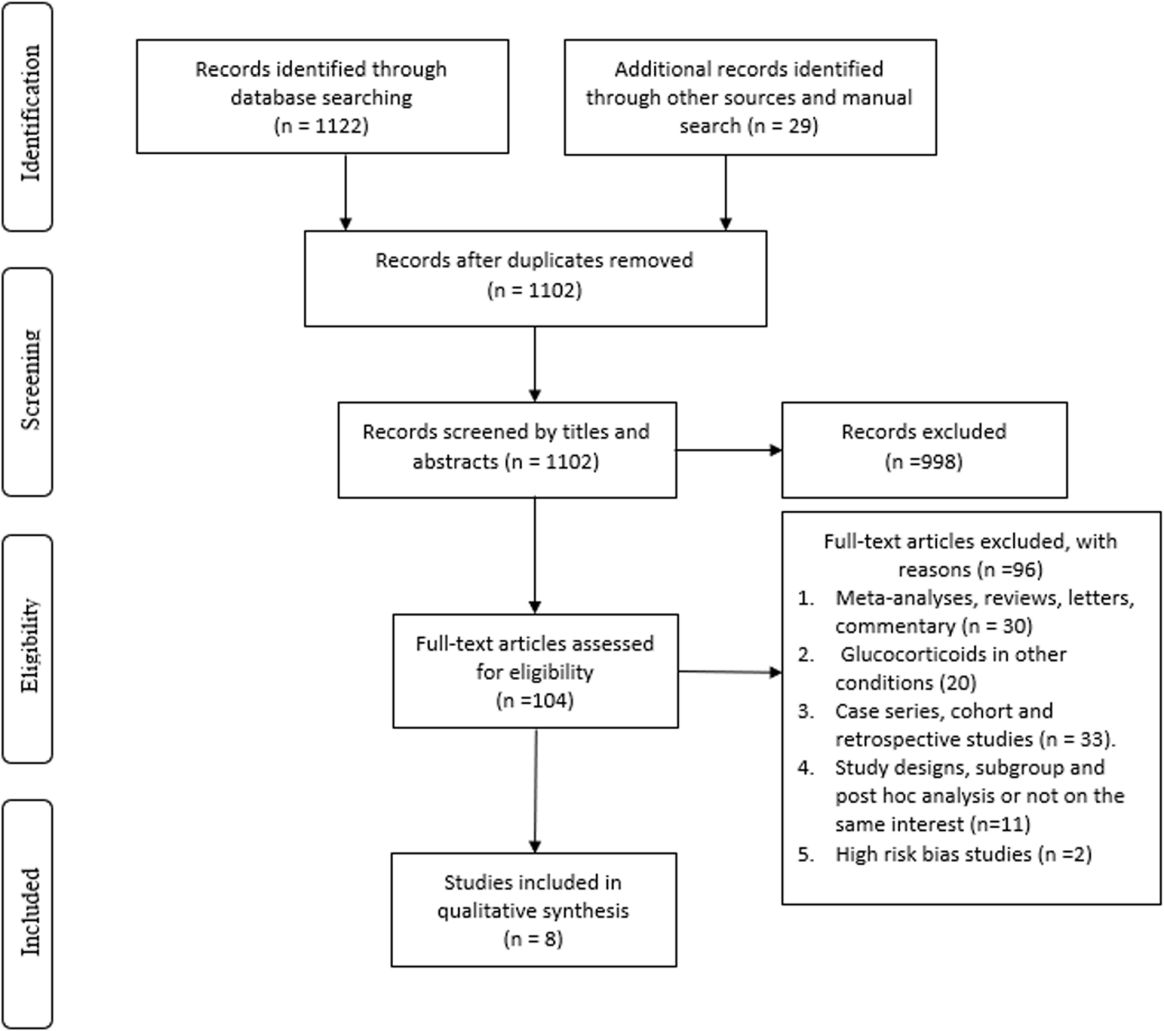
Flow chart of literature search and study selection

After review of electronic databases, we included 8 RCTs totaling 1091 patients with a mean age 57 ± 16 years, and 56.2% were male [4, 10–16]. Figure 1 illustrates the search process and study selection. Six trials initiated glucocorticoids treatment within 7 days of ARDS onset, while 2 trials initiated glucocorticoid treatment after 7 days of ARDS onset [11, 13]. Glucocorticoids were administered for a total of 7–14 days in five of the included trials. Two trials used extended duration of glucocorticoids (28–32 days) [12, 13]. In addition, one trial administered high-dose methylprednisolone (120 mg/Kg divided on 4 doses) for only 24 h [16]. Two studies were excluded from the final analysis due to high risk of bias concerning the blinding of participants and investigators [17, 18]. Table 1 explains the characteristics of included studies while Table 2 explains the demographic and clinical characteristics of the included patient populations in each study.

**Table 1:** Characteristics of included studies

Study name (first author and year)	Study design	Study groups and patients’ number	Inclusion criteria	Treatment regimen	Duration of treatment	Follow-up duration
Bernard 1987	Multicenter randomized controlled trial	Total patients, 99 Steroids, 50 Placebo, 49	Adult patients having ARDS withPaO2 ≤ 7 0mmhg (with FiO2 at least 40%) or PaO2/PAO2 ≤ 0.3; bilateral diffuse infiltrates on chest radiography, PAWP ≤ 18mmhg regardless of PEEP level.	-Treatment started after ARDS onset. Methylprednisolone 30 mg every 6 h (4 doses only).	24 h	Until death or for 45 days
Meduri 1998	Multicenter randomized controlled trial	Total Patients, 24 Steroids, 16 Placebo, 8	Adult patients with hypoxemic respiratory failure diagnosed with ARDS, who were on mechanical ventilation for at least 7 days, with LIS of 2.5 or greater and less than 1-point reduction from day 1 of ARDS onset.	-Treatment started after 7 days of ARDS onset. -Methylprednisolone 2 mg/kg per day (2 mg/kg from day 1 to day 14; 1 mg/kg from day 15 to 21; 0.5 mg/kg from day 22 to day 28; 0.25 mg/day from day 28 to day 32).	32 days	Hospital length of stay.
Confalonieri 2005	Multicenter randomized controlled trial	Total patients, 46 Steroids, 22 Placebo, 23	Patients with clinical and radiographic evidence of pneumonia with bilateral or multi-lobar involvement and PaO2/FiO2 ratio less than 250.	-Treatment started after diagnosis. -Hydrocortisone 200 mg bolus followed by an infusion of 10 mg/h.	7 days	60 days
Annane 2006	Multicenter randomized controlled trial	Total patients, 177 Steroids, 85 Placebo, 92	Patients with septic shock and ARDS; bilateral infiltrates on chest radiography; PaO2/FiO2 ≤ 200, PAWP ≤ 18 mmhg; no left atrial hypertension.	-Treatment started after randomization which occurred within 8 h of disease onset. -Hydrocortisone 50 mg every 6 h and 9 alpha fludrocortisone 50 milligram orally once a day.	7 days	28 days
Steinberg 2006	Multicenter randomized control trial	Total patients, 180 Steroids, 89 Placebo, 91	Adult patients who had ARDS and mechanically ventilated for 7 to 28 days. PaO2/FiO2 ≤ 200 mmhg.	-Treatment started after 7 to 28 days of ARDS onset. -Methylprednisolone: bolus 2 mg/kg followed by 0.5 mg/kg every 6 h for 14 days and then 0.5 mg/kg every 12 hours for 7 days and then tapering over 4days.	21-25 days.	60 days
Meduri 2007	Multicenter randomized control trial	Total patients, 91 Steroids, 63 Placebo, 28	Adult intubated patients with early ARDS (≤ 72 h) defined by the American-European Consensus definition.	-Treatment started within 72 h of ARDS onset. -Methylprednisolone bolus dose of 1 mg/kg followed by an infusion of 1 mg/kg per day from day 1 to day 14; 0.5 mg/kg per day from day 15 to day 21; 0.25 mg/kg per day from day 22 to day 25; and 0.125 mg/kg per day from day 26 to day 28.	Up to 28 days	Hospital length of stay.
Tongyoo 2016	Single-center randomized controlled trial	Total patients, 197 Steroids, 98 Placebo, 99	Adult patients with severe sepsis or septic shock, intubated with ARDS (according to criteria of ARDS by the American European Consensus or by the berlin criteria as moderate to severe ARDS)	-Randomization within 12 h of ARDS onset. -Hydrocortisone 50 mg every 6 h for 7 days	7 days	28 days
Villar 2020	Multicenter randomized control trial	Total patients, 277 Steroids, 139 Placebo, 138	Adult intubated patients with acute onset of ARDS according to criteria of ARDS by the American European Consensus or by the berlin criteria as moderate to severe ARDS.	Dexamethasone 20 mg daily from day 1 to day 5, then 10 mg daily from day 6 to day 10.	10 days	60 days

*ARDS* acute respiratory distress syndrome, *PaO2* partial pressure pf arterial oxygen, *PAWP* pulmonary artery wedge pressure, *PEEP* positive end expiratory pressure, *LIS* lung injury score, *FiO2* fraction of inhaled oxygen, *APACHE* acute physiologic and chronic health evaluation, *MMHG* millimeter of mercury, *MG* milligram, *KG* kilogram

**Table 2:** Baseline clinical characteristics of patients

Study	Study groups	Total number	Age	Male No. (%).	APACHE III score	Respiratory rate	PaO2/FiO2	PEEP
Bernard 1987	Steroid	50	55 ± 2	NA	NA	NA	NA	8 ± 1
	Control	49	56 ± 2	NA	NA	NA	NA	7 ± 1
Meduri 1998	steroid	16	47 ± 3.9	5 (31%)	58(14)	NA	161(14)	12(1.2)
	control	8	51 ± 6.6	4 (50%)	55(16)	NA	141(19)	14(1.7)
Confalonieri 2005	steroid	23	60.4 ± 17.3	17 (74%)	17.2 ± 4.1	NA	141 ± 49	NA
	control	23	66.6 ± 14.7	15 (65%)	18.2 ± 4	NA	178 ± 58	NA
Annane 2006	steroid	85	61 ± 16	56 (66 %)	NA	18.5 ± 3	104 ± 42	6.8 ± 2.7
	control	92	59 ± 18	65 (70%)	NA	17.9 ± 3.1	108 ± 45	7.4 ± 3
Steinberg 2006	steroid	89	49 ± 19	40 (45%)	87.6 ± 27.5	NA	126 ± 42	12.9 ± 5.6
	control	91	49.2 ± 16.5	58 (64%)	84.6 ± 29.4	NA	126 ± 40	12.3 ± 4.7
Meduri 2007	steroid	63	50.1 ± 15.3	34 (54%)	60.2 ± 20.2	NA	118.4 ± 51.2	13 ± 5
	control	28	53.2 ± 15.3	13 (46%)	57.9 ± 21	NA	125.9 ± 38.6	11.2 ± 4
Tongyoo 2016	steroid	98	64.5 ± 17.3	50 (51%)	21.7 ± 5.7	NA	175.4 ± 6.9	7.3 ± 3
	control	99	64.3 ± 16	51 (52%)	21.9 ± 5.7	NA	172.4 ± 6.7	6.8 ± 2.5
Villar 2020	steroid	139	56 ± 14	96 (69%)	NA	23(5)	142.4 ± 37.3)	12.6 ± 2.7
	control	138	58 ± 15	95 (69%)	NA	23(5)	143.5 ± 33.4	12.5 ± 2.6

Data are provided number and percent (%) or mean ± SD. *PaO2* partial pressure pf arterial oxygen, *PEEP* positive end expiratory pressure, *FiO2* fraction of inhaled oxygen, *APACHE* acute physiologic and chronic health evaluation

**Fig. 2:**
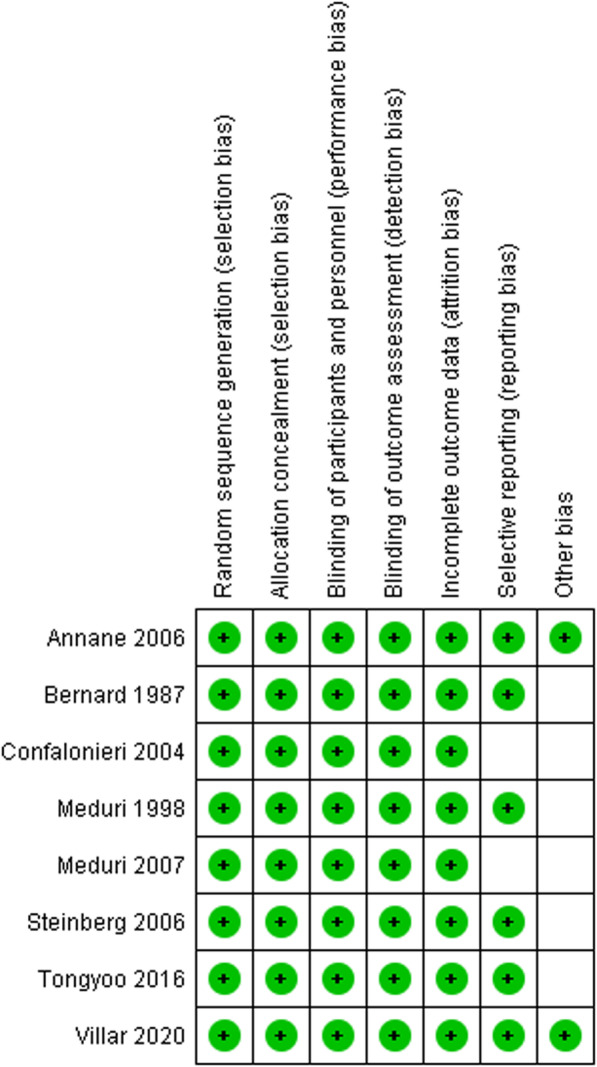
Risk of bias assessment based on authors’ judgment for each of the included RCTs. Blank items indicate an unclear risk of bias

Figure 2 explains the results of the quality assessment based on authors’ judgment.

### Clinical outcomes

#### Hospital and ICU-mortality

**Fig. 3:**
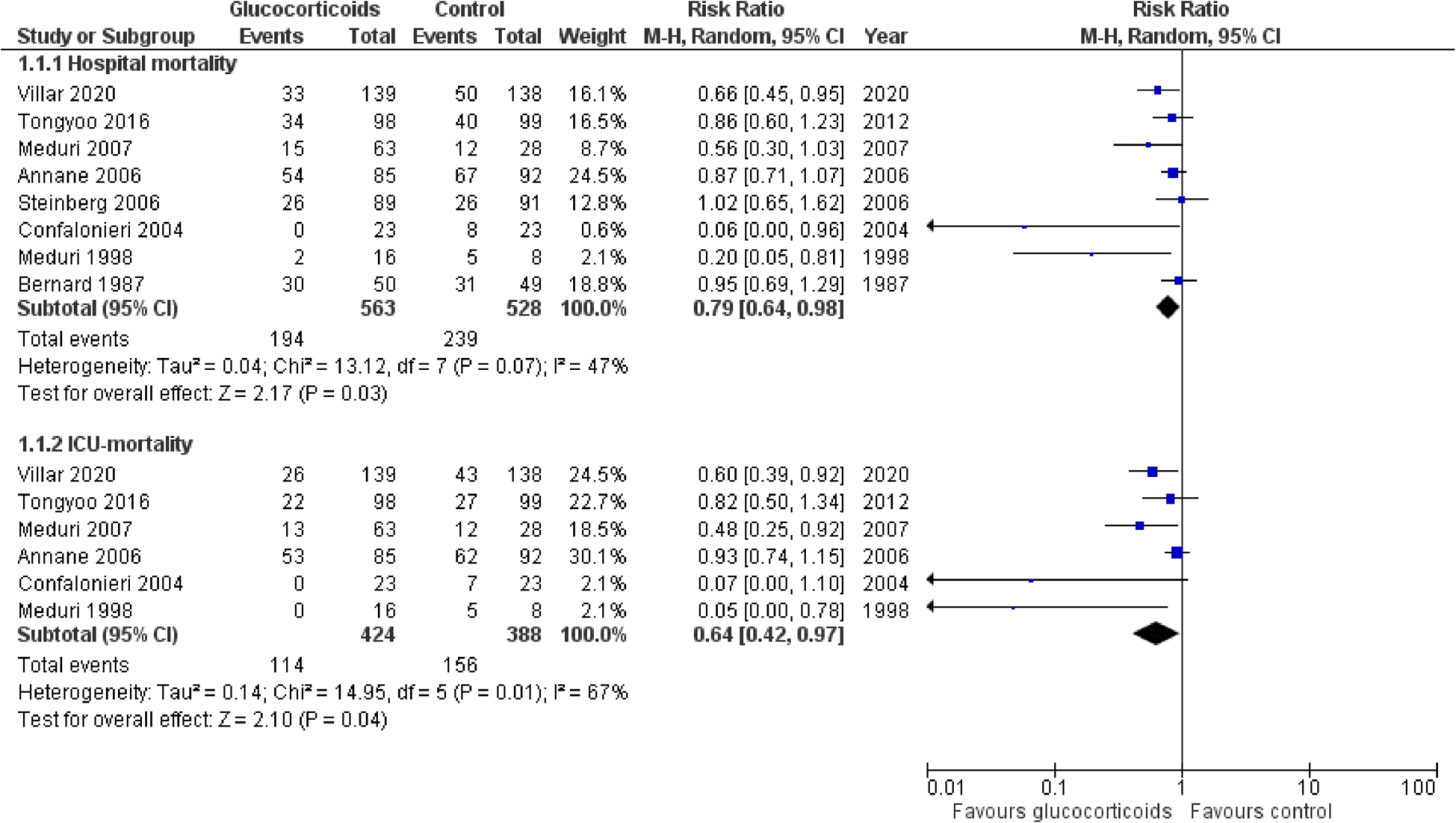
Forest plot for hospital and ICU mortality

Use of glucocorticoids was associated with a significant reduction of hospital mortality (RR 0.79; 95% CI 0.64–0.98; *P* = 0.03; *I*^2^ = 47%) and ICU mortality (RR 0.64; 95% CI 0.42–0.97; *P* = 0.04, *I*^2^ 67%) (Fig. 3). Sensitivity analysis with sequential trial removal revealed consistent results.

Subgroup analysis showed that there was no hospital mortality benefit with late administration (more than 7 days of ARDS onset) of glucocorticoids (RR 0.52; 95% CI 0.11–2.52; *P* = 0.42; 2 studies, 204 patients) while mortality benefit remained significant with early glucocorticoids administration (RR 0.80; 95% CI 0.65–0.98; *P* = 0.03; 6 studies, 887 patients). Further subgroup analysis revealed that there was no significant difference in hospital mortality in studies that used glucocorticoids without lung protective ventilation (RR 0.79; 95% CI 0.58–1.07; *P* = 0.12; 6 studies, 667 patients), while the mortality reduction remained significant in studies incorporating a lung protective ventilation strategy (RR 0.75; 95% CI 0.58–98; *P* = 0.04; 2 studies, 474 patients). Meta-regression analysis did not suggest any effects of the study-level covariates on hospital mortality; however, prolonged duration of glucocorticoid treatment and higher PEEP were associated with decreased ICU mortality (P < 0.05) (Supplementary Figure 1 & 2).

**Fig. 4:**
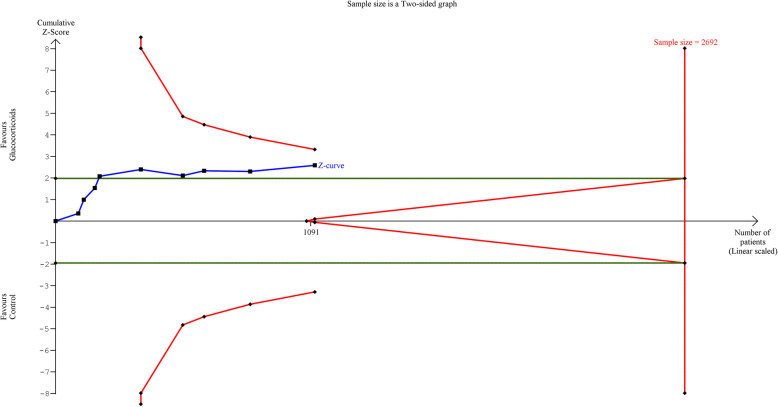
Trial sequential analysis for hospital mortality. The diversity-adjusted information size (sample size) is 2692 patients. The cumulative Z-line (blue line with small black squares representing each trial) crosses the alpha monitoring boundary (horizontal green line) indicating statistical significance for the efficacy of glucocorticoids. However, The Z-line failed to cross the TSA boundary (concave red line), and since the required sample size was not reached, there is lack of firm evidence supporting improved hospital mortality in the glucocorticoids group

In a trial sequential analysis for hospital mortality, the cumulative Z-curve crossed the Alpha boundary of significance, indicating sufficient statistical significance favoring glucocorticoids over the control group. However, since the cumulative Z-curve failed to cross the TSA boundary and the diversity-adjusted information size (sample size) calculated (2692 patients) was not reached, the conclusion is insufficient, and further studies are needed (Fig. 4). Similarly, regarding ICU mortality, the conclusion was insufficient, and further studies are needed (Supplementary Figure 3). Similar conclusions were obtained upon performing sensitivity analyses with 15% and 25% RRR in mortality.

### Number ventilator-free days at day 28

**Fig. 5:**
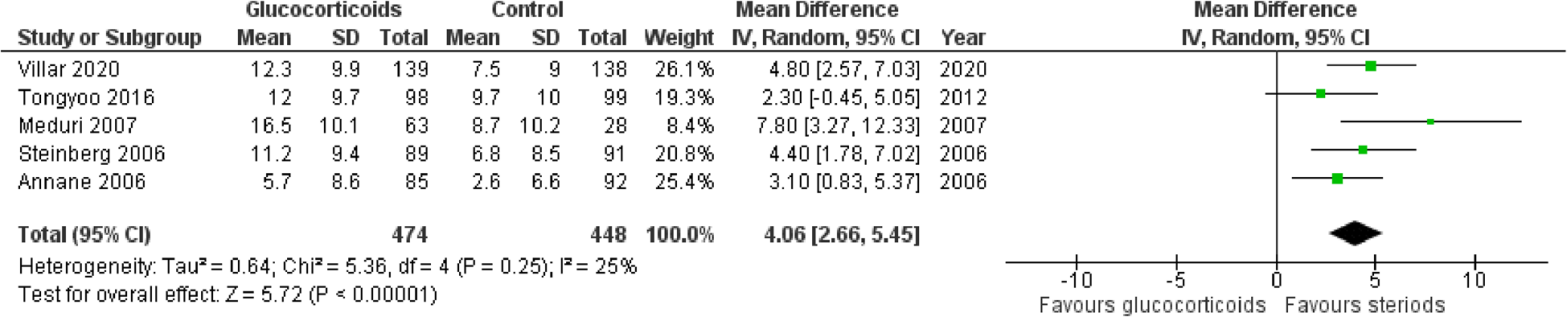
Forest plot for number of ventilator-free days at day 28

There was a significant increase in the number of ventilator-free days at day 28 in patients treated with glucocorticoids in comparison to the control group (MD 4.06 days; 95% CI 2.66–5.45; *P* < 0.01; *I*^2^ = 25%) (Fig. 5).

### Adverse events

**Fig. 6:**
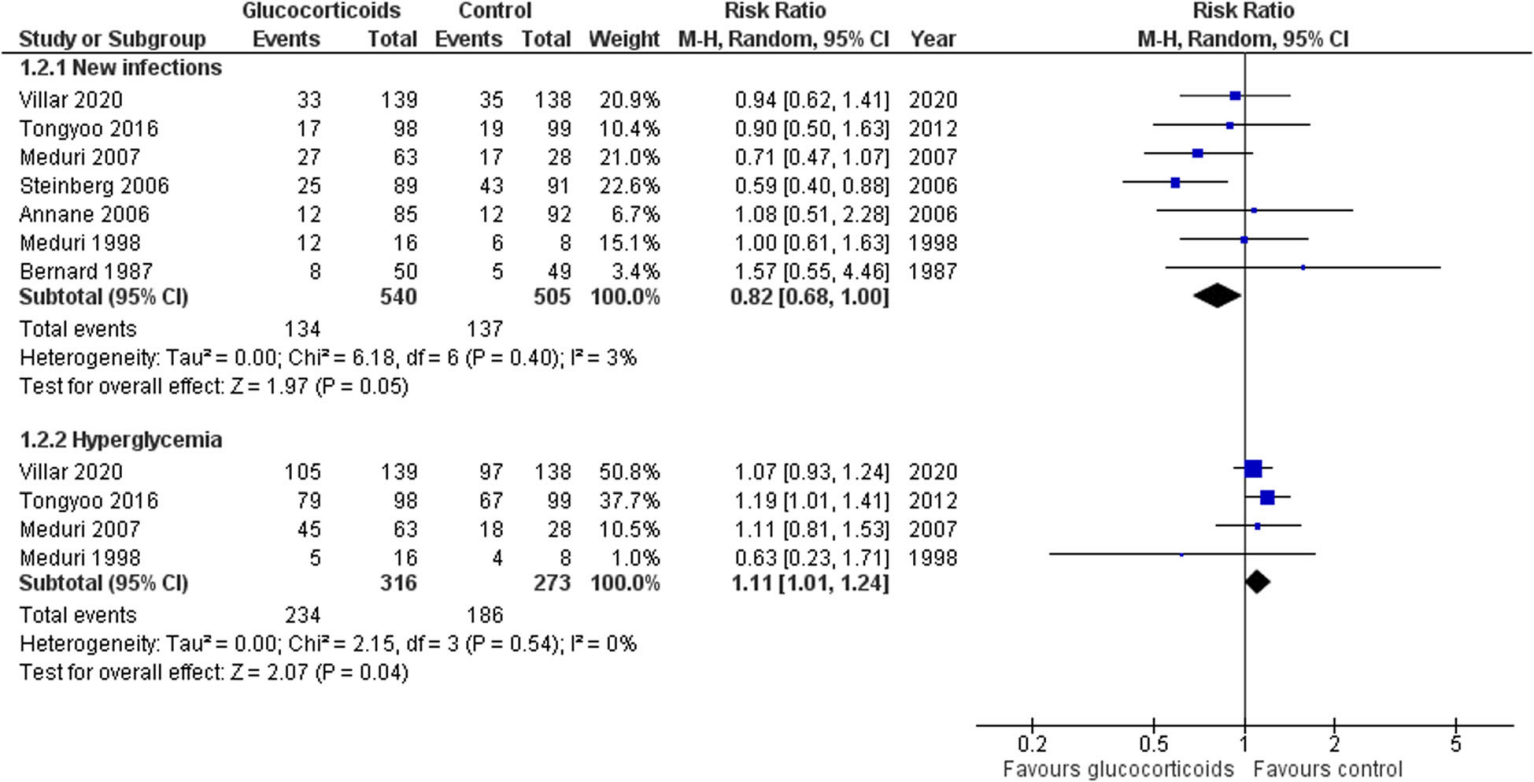
Forest plot for adverse events, infection, and hyperglycemia

Use of glucocorticoids was not associated with an increased risk of hospital-acquired infections (RR 0.82; 95% CI 0.68–1.00; *P* = 0.05; *I*^2^ = 3%) but was associated with an increased risk of hyperglycemia (RR 1.11; 95% CI 1.01–1.24; *P* = 0.04; *I*^2^ = 0%) (Fig. 6). Meta-regression analysis did not suggest any covariates effects on the adverse events.

## Discussion

In our meta-analysis, use of glucocorticoids in patients with ARDS was associated with a significant reduction in hospital and ICU mortality and duration of mechanical ventilation. While there was no increased risk of hospital-acquired infections with glucocorticoid use, there was an increased risk of hyperglycemia. In trial sequential analysis, these findings could be secondary to a false-positive (type I) error, and further studies are needed for sufficient evidence as the required sample size was not reached by the available RCTs.

Current guidelines of American Thoracic Society/European Society of intensive care medicine/Society of Critical Care Medicine have strong recommendations for the use of low tidal volume (4–8 ml/kg of ideal body weight), limiting inspiratory pressure (plateau pressure < 30 cm H_2_O), and prone positioning in moderate-to-severe ARDS. Furthermore, the use of recruitment maneuvers and higher PEEP strategies have conditional recommendations in patients with moderate-to-severe ARDS. In addition, glucocorticoids have a conditional recommendation in early moderate-to-severe ARDS, and their use is still controversial [6, 19].

There are three distinct phases in the development of ARDS including exudative, proliferative, and fibrotic phases [1]. As lung fibrosis is associated with increased duration of mechanical ventilation and increased rates of mortality, steroids are considered a potent anti-inflammatory agent that can attenuate the inflammatory process and subsequently decrease further lung injury and fibrosis [1].

A recently published randomized controlled trial revealed that early use of dexamethasone in patients with moderate-to-severe ARDS was associated with a significant reduction in mortality and duration of mechanical ventilation [4]. Similar results were noticed in patients with sepsis or septic shock with moderate-to-severe ARDS treated with methylprednisolone in comparison to placebo [10]. In these two recent trials, glucocorticoid use was evaluated with lung protective mechanical ventilation and low tidal volume, as opposed to the other trials conducted before 2005 where low tidal volumes were not implemented in the trial protocols. This strategy which limits tidal volume to 4–8 ml/kg of ideal body weight and alveolar pressure to less than 30 cm H_2_O showed a significant reduction in mortality and increased number of ventilator-free days at day 28 [20].

In our subgroup analysis, we found that there was no mortality benefit in studies that evaluated glucocorticoids without a lung-protective ventilation strategy likely secondary to worsening lung injury. High tidal volumes delivered to an already injured lung may worsen lung injury leading to alveolar rupture, air leaks, and barotrauma with worse clinical outcomes [20–22]. Furthermore, we found that late administration of glucocorticoids (after 7 days of ARDS onset) was not associated with improved outcomes despite lower risk ratio (0.52) but with a high *p* value, a finding that is limited by the low number of patients and studies in this subgroup (2 studies, 204 patients). However, this supports the concept that steroids exert their action through downregulation of the inflammatory response and decrease alveolar capillary permeability which occurs early in the exudative phase and is linked to lung injury [1]. In exploratory meta-regression, we found that patients who were treated with prolonged duration of glucocorticoid administration and received higher PEEP had lower ICU but not hospital mortality; a finding that is limited by the low number of studies that reported ICU mortality and needs to be examined in further trials.

Our analysis revealed a 21% risk reduction in hospital mortality among ARDS patients treated with glucocorticoids with a number needed to treat of nine patients to prevent one death. In addition, there was a 4-day increase in the number of ventilator-free days at day 28. Despite these favorable outcomes, there was no increased risk of hospital acquired infections; in contrast, our analysis showed a tendency toward reduction of acquired infections, a finding that could be explained by the decreased duration of mechanical ventilation and subsequently ICU length of stay. However, these findings should be interpreted cautiously until confirmed in further larger studies.

In order to examine the strength of the evidence and whether more randomized controlled trials are needed for sufficient conclusion regarding mortality benefit, we performed a trial sequential analysis to guard against false positive (type I) or false negative (type II) errors. While the mortality benefit reached statistical significance, based on our analysis, the mortality benefit could be secondary to a false positive (type I) error, and the evidence is insufficient as the sample size required for detection of 20% RRR in mortality between the two groups while avoiding statistical errors is 2692 patients that was not reached by the available data (1091) patients were included in our analysis). Further, well-controlled randomized clinical trials are required for a strong conclusion about the efficacy of steroids in managing ARDS patients. Additionally, the focus should be on the type, dose, and duration of glucocorticoids therapy as we included studies that evaluated different glucocorticoids with variable dosages and durations. However, until further studies are performed, the significant risk reduction and the low number needed to treat may justify the use of glucocorticoids in patients with ARDS, especially those with an underlying etiology similar to patients enrolled in the included RCTs (sepsis, septic shock, and pneumonia).

Our results are consistent with previously published meta-analyses. However, we included the recently published trial and only included patients with established ARDS, and we excluded studies with high risk of bias as well as retrospective studies which were included in previous reviews [23, 24]. Furthermore, we were able to perform subgroup and meta-regression analyses based on study-level covariates. In addition, we conducted trial sequential analysis to examine the strength of the evidence and concluded that further studies are needed for a strong and firm evidence of glucocorticoids efficacy in ARDS patients with paying special attention of the duration, dose, and timing of glucocorticoids administration.

Our results are non-generalizable to patients with ARDS secondary to coronavirus disease-2019 (COVID-19) and other viral pneumonias such as H1N1 influenza. To date, there is only one retrospective study that examined outcomes of COVID-19 patients treated with steroids. The study by Wu et al. found lower risk of death (hazard ratio 0.38; 95% CI 0.20–0.72; *p* = 0.003) in patients with ARDS treated with methylprednisolone [25]. In addition, a non-peer reviewed article was published recently and reported reduction in the duration of supplemental oxygen and improved radiographic findings in 26 patients with severe COVID-19 but it is unknown how many patients had ARDS in this cohort [26]. Since we lack patient-level data and the information are missing in the published literature, we were unable to perform a subgroup analysis for patients with viral etiology of ARDS. However, it is known that glucocorticoids are associated with worse outcomes in patients with ARDS secondary to H1N1 influenza virus as demonstrated by previous cohort studies and meta-analyses [27–29]. Based on the current available evidence for the management of COVID-19 patients, Society of Critical Care Medicine and European Society of intensive care medicine guidelines have recommended against the use of corticosteroids in mechanically ventilated patients without ARDS and issued a weak recommendation for the use of low dose steroids (hydrocortisone 200 mg per day) in those with ARDS and/or refractory septic shock [30].

## Limitations

Our study has several limitations. There is significant advancement in critical care management between older and modern studies as most of the studies did not adopt lung-protective ventilation. Second, we included RCTs that investigated different types and dosages of glucocorticoids with various durations. Third, as we lack patient-level data, we could not perform analyses based on the severity and underlying etiology of ARDS.

## Conclusion

Among patients with ARDS, use of glucocorticoids is associated with significant reduction in mortality and duration of mechanical ventilation without an increased risk of infection but with an increased incidence of hyperglycemia. In our trial sequential analysis, we revealed that the evidence is insufficient from the available RCTs, and further studies are required for a firm conclusion with guarding against possible statistical errors.

## Supplementary information

**Additional file 1: Supplementary Figure 1.** Regression of duration of glucocorticoid treatment on ICU mortality. Longer duration was associated with lower rates of ICU mortality (*P* < 0.05).

**Additional file 2: Supplementary Figure 2.** Regression of PEEP on ICU mortality. Higher PEEP was associated with lower rates of ICU mortality (*P* < 0.05).

**Additional file 3: Supplementary Figure 3.** Trial sequential analysis for ICU-mortality. The diversity-adjusted information size (sample size) is 3,244 patients. The cumulative Z-line (blue line with small black squares representing each trial) crosses the alpha monitoring boundary (horizontal green line) indicating statistical significance for the efficacy of glucocorticoids. However, The Z-line failed to cross the TSA boundary (concave red line), and since the required sample size was not reached, there is lack of firm evidence supporting improved hospital mortality in the glucocorticoids group.

## Data Availability

Data and materials are available and can be presented upon request.
