# Supplementary figures and images for: Use of glucocorticoids in patients with acute respiratory distress syndrome: a meta-analysis and trial sequential analysis
Source: J Intensive Care. 2020 Jun 30;8:43. doi: 10.1186/s40560-020-00464-1 (PMC7324774; doi:10.1186/s40560-020-00464-1)

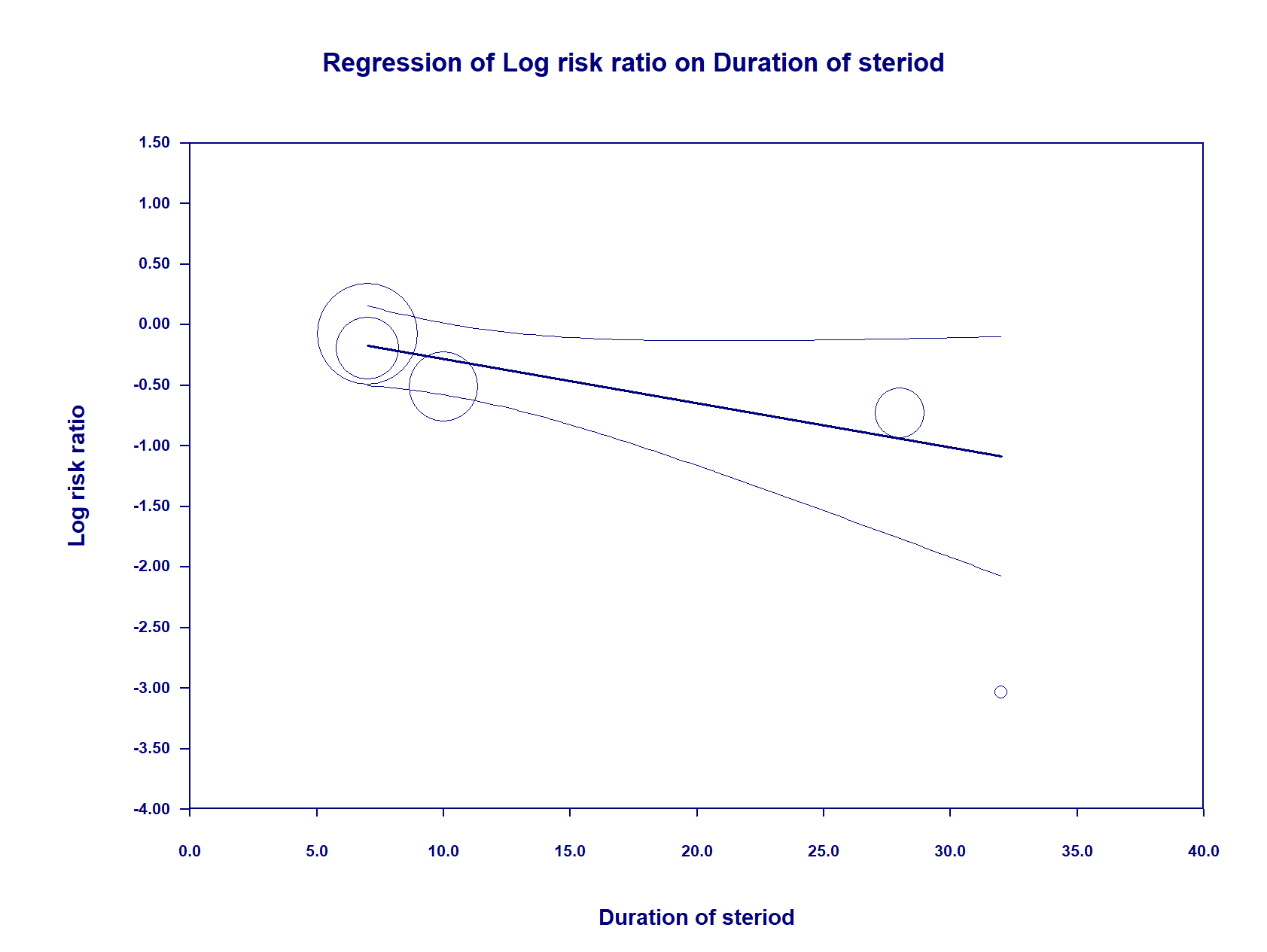

Supplement: Supplementary file 1 — Additional file 1: Supplementary Figure 1. Regression of duration of glucocorticoid treatment on ICU mortality. Longer duration was associated with lower rates of ICU mortality (P < 0.05). [file 40560_2020_464_MOESM1_ESM.png]

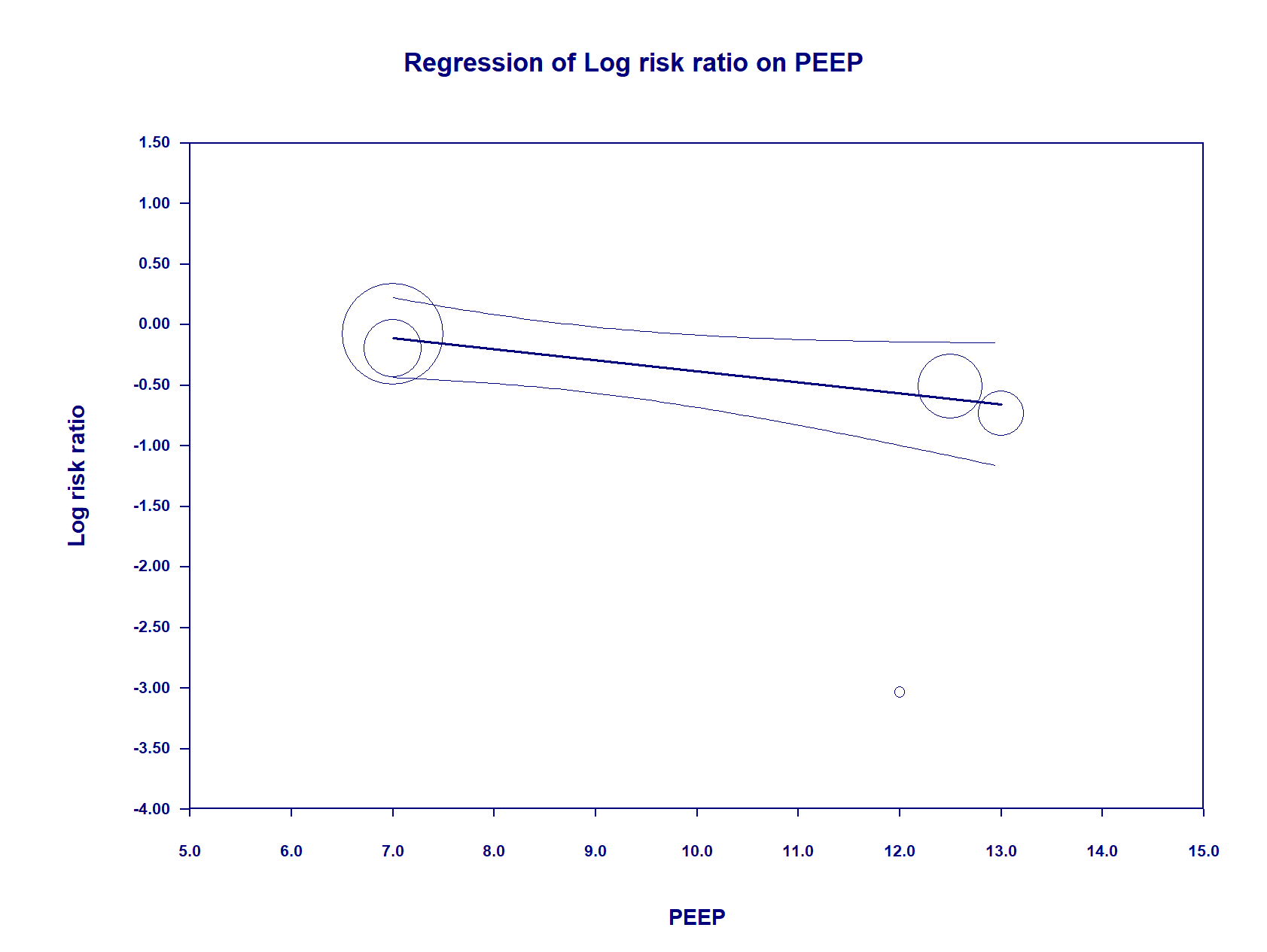

Supplement: Supplementary file 2 — Additional file 2: Supplementary Figure 2. Regression of PEEP on ICU mortality. Higher PEEP was associated with lower rates of ICU mortality (P < 0.05). [file 40560_2020_464_MOESM2_ESM.png]

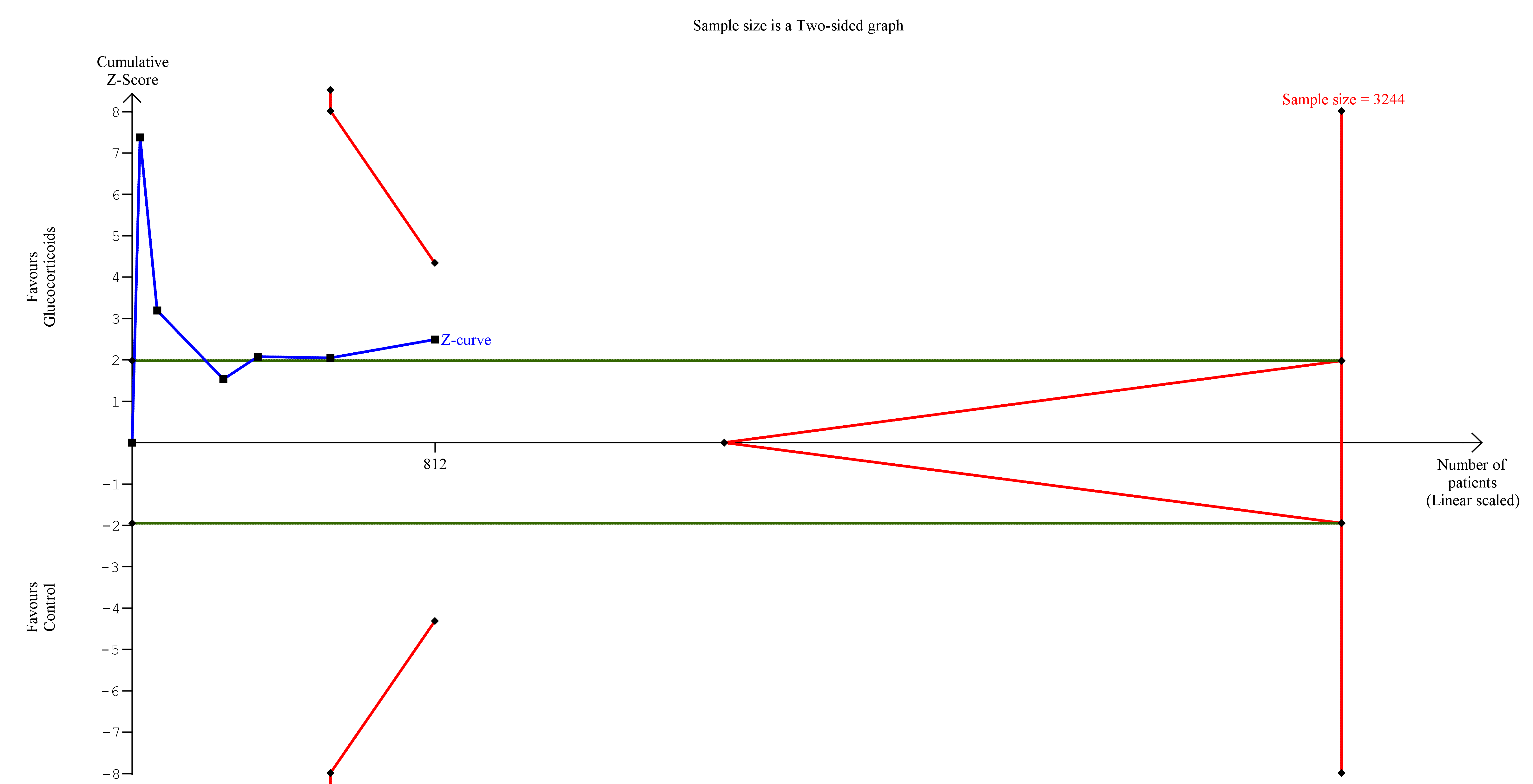

Supplement: Supplementary file 3 — Additional file 3: Supplementary Figure 3. Trial sequential analysis for ICU-mortality. The diversity-adjusted information size (sample size) is 3,244 patients. The cumulative Z-line (blue line with small black squares representing each trial) crosses the alpha monitoring boundary (horizontal green line) indicating statistical significance for the efficacy of glucocorticoids. However, The Z-line failed to cross the TSA boundary (concave red line), and since the required sample size was not reached, there is lack of firm evidence supporting improved hospital mortality in the glucocorticoids group. [file 40560_2020_464_MOESM3_ESM.tif]
